# Randomized Trial of Community Treatment With Azithromycin and Ivermectin Mass Drug Administration for Control of Scabies and Impetigo

**DOI:** 10.1093/cid/ciy574

**Published:** 2018-07-07

**Authors:** Michael Marks, Hilary Toloka, Ciara Baker, Christian Kositz, James Asugeni, Elliot Puiahi, Rowena Asugeni, Kristy Azzopardi, Jason Diau, John M Kaldor, Lucia Romani, Michelle Redman-MacLaren, David MacLaren, Anthony W Solomon, David C W Mabey, Andrew C Steer

**Affiliations:** 1 Clinical Research Department, Faculty of Infectious and Tropical Diseases, London School of Hygiene & Tropical Medicine, United Kingdom; 2 Hospital for Tropical Diseases, University College London Hospitals NHS Trust, United Kingdom; 3 Atoifi Adventist Hospital, Malaita Province, Solomon Islands; 4 Group A Streptococcal Research Group, Murdoch Children’s Research Institute, Melbourne, Victoria, Australia; 5 National Referral Hospital, Honiara, Solomon Islands; 6 Kirby Institute, University of New South Wales, Sydney; 7 College of Medicine and Dentistry, James Cook University, Cairns, Queensland, Australia; 8 Centre for International Child Health, University of Melbourne, Victoria, Australia; 9 Department of General Medicine, Royal Children’s Hospital, Melbourne, Victoria, Australia

**Keywords:** scabies, neglected tropical diseases, ivermectin, impetigo, antimicrobial resistance

## Abstract

**Background:**

Scabies is a public health problem in many countries, with impetigo and its complications important consequences. Ivermectin based mass drug administration (MDA) reduces the prevalence of scabies and, to a lesser extent, impetigo. We studied the impact of co-administering azithromycin on the prevalence of impetigo and antimicrobial resistance.

**Methods:**

Six communities were randomized to receive either ivermectin-based MDA or ivermectin-based MDA co-administered with azithromycin. We measured scabies and impetigo prevalence at baseline and 12 months. We collected impetigo lesions swabs at baseline, 3 and 12 months to detect antimicrobial resistance.

**Results:**

At baseline, scabies and impetigo prevalences were 11.8% and 10.1% in the ivermectin-only arm and 9.2% and 12.1% in the combined treatment arm. At 12 months, the prevalences had fallen to 1.0% and 2.5% in the ivermectin-only arm and 0.7% and 3.3% in the combined treatment arm. The proportion of impetigo lesions containing *Staphylococcus aureus* detected did not change (80% at baseline vs 86% at 12 months; no significant difference between arms) but the proportion containing pyogenic streptococci fell significantly (63% vs 23%, *P* < .01). At 3 months, 53% (8/15) of *S. aureus* isolates were macrolide-resistant in the combined treatment arm, but no resistant strains (0/13) were detected at 12 months.

**Conclusions:**

Co-administration of azithromycin with ivermectin led to similar decreases in scabies and impetigo prevalence compared to ivermectin alone. The proportion of impetigo lesions containing pyogenic streptococci declined following MDA. There was a transient increase in the proportion of macrolide-resistant *S. aureus* strains following azithromycin MDA.

**Clinical Trials Registration:**

clinicaltrials.gov (NCT02775617).

## Teaser

Scabies is a major cause of impetigo. We assessed the effect on impetigo prevalence of adding azithromycin to ivermectin mass drug. The decrease in impetigo did not differ between communities treated with ivermectin and communities treated with ivermectin and azithromycin.

Scabies is a major public health problem in many tropical countries [1]. As well as the direct consequences of infestation, scabies leads to an increased risk of secondary bacterial skin disease (impetigo), mostly due to *Staphylococcus aureus* and *Streptococcus pyogenes* [2], due to breaks in the skin and possibly down-regulation of complement by *Sarcoptes scabei* [3]. Skin infections, especially those due to *S. pyogenes*, can result in more serious disease, including bacteremia, glomerulonephritis, and possibly rheumatic heart disease [1, 4–6]. In 2017 scabies was formally recognized as a neglected tropical disease (NTD) by the World Health Organization (WHO), leading to increased interest in strategies for controlling scabies and its associated morbidity.

Mass drug administration (MDA) has been demonstrated to be effective as a control measure for scabies, through single-arm studies using permethrin or ivermectin [7–10] and, recently, a comparative trial in Fiji, which demonstrated ivermectin was superior to permethrin [11]. In these studies, community-wide treatment for scabies, without antibacterial therapy, led to substantial reductions in impetigo.

Given the ongoing burden of impetigo and its complications in these settings, it is reasonable to consider whether the addition of an antibacterial agent may be beneficial. The macrolide azithromycin is a potential candidate for this role, because it has good activity against *S. pyogenes* and *S. aureus*. Because of its long half-life and low toxicity, it is recommended by WHO for mass drug administration for control of trachoma, and eradication of yaws [12, 13].

Any benefit from community-wide use of antimicrobial agents needs to be weighed against the risk of promoting the selection of antimicrobial resistant organisms. A number of studies have assessed the impact of azithromycin MDA on nasopharyngeal or oropharyngeal carriage of azithromycin resistant bacteria [14–18], but none have assessed the impact on organisms isolated from impetigo lesions.

As in many Pacific Island nations, the prevalence of scabies and impetigo is high in the Solomon Islands [7, 19–21]. Yaws and trachoma have also been found at high levels in the Solomon Islands [22–24]. This co-endemicity has provided a rationale to consider co-administration of ivermectin and azithromycin. Previous studies suggest that co-administration is safe compared to individual use of the 2 agents [25, 26].

We conducted a community randomised trial to assess whether adding azithromycin to ivermectin-based MDA for scabies had an additional impact on the prevalence of impetigo at 12 months or on antimicrobial resistance of Gram-positive bacteria isolated from impetigo lesions.

## METHODS

### Study Setting and Recruitment

This was a community randomised open label study conducted in Malaita province of the Solomon Islands. Six communities were randomized to one of 2 arms: an ivermectin arm or a combined-treatment arm. We selected communities that were isolated from each other to reduce contamination between the 2 study arms.

All residents living in selected communities were eligible to participate. Community engagement and education were conducted by the study team prior to commencement of the study. Written informed consent was obtained from adults and from the parent or guardian of children. Assent was also obtained from children who were able to provide it.

### Data Collection

Study visits took place at 3 timepoints. At baseline, participants were seen for enrollment, initial data collection and treatment. At 3 months, we reexamined children (aged ≤12 years) in each community to allow for collection of swabs to monitor for antimicrobial resistance (see below); this age group was selected as they were anticipated to have the highest prevalence of impetigo. At the 12-month follow-up visit, we again aimed to examine all participating residents in participating communities. Prior to visits at both baseline and 12 months, the study team conducted a village census. At baseline and 12 months, participants underwent a standardized examination by an experienced clinician (MM) with data recorded on the presence or absence of any skin lesions, their location, and whether they were consistent with scabies, impetigo, or another diagnosis. The clinical diagnosis of scabies was based on the morphology (burrows, papules, nodules, vesicles) and distribution of rash alongside the presence of pruritus or evidence of excoriation. Active impetigo was diagnosed on the basis of discrete papular, pustular, or ulcerative lesions with associated erythema, crusting, bullae, or frank pus [27]. The severity of scabies and impetigo was classified as previously described [19]. Data were collected directly into Android smartphones using the OpenDataKit software package [28].

### Treatment

Treatment was offered to all participating members of the community and was directly observed by the study team. In the ivermectin arm we administered ivermectin MDA at baseline. In the combined treatment arm we co-administered ivermectin and azithromycin MDA at baseline. Ivermectin MDA consisted of a single oral dose of ivermectin (200 μg/kg) determined by body weight. In individuals with a contra-indication to ivermectin (pregnancy, breast-feeding, weight <15 kg) topical permethrin was offered instead. Individuals clinically diagnosed with scabies at baseline were offered a second dose of ivermectin (or second application of topical permethrin) at 7 days [11]. Azithromycin MDA consisted of a single oral dose of azithromycin (30 mg/kg, max 2 gm) determined by body weight [29, 30]

### Sample Collection and Analysis

To assess changes in antimicrobial resistance, we aimed to collect swabs from approximately 40 active impetigo lesions in children (≤12 years) per treatment arm at baseline (equivalent to approximately one third of our anticipated cases of impetigo at baseline). At 3 months, swabs were collected from all children with active impetigo. Finally, at 12 months we again aimed to collect swabs from all individuals with active impetigo. We collected swabs from a single lesion in each individual. A sterile cotton-tipped swab was rolled across pus or exudate from active impetigo lesions and placed inside a dry-transport tube, then shipped at ambient temperature within 7 days [31]. Swabs were sent to the Murdoch Children’s Research Institute, Melbourne, Australia, where they were streaked onto horse blood agar plates and incubated at 37° C in 5% CO_2_. Plates were reviewed at 24 hours and purity plating performed. Beta-hemolytic streptococcal colonies were grouped by latex agglutination (Pro-Lab Diagnostics, Richmond Hill, Canada). *S. aureus* colonies were detected using a latex slide agglutination test (Oxoid, United Kingdom). Antimicrobial sensitivity testing was performed using VITEK 2 (bioMérieux Inc., Durham, NC). We inferred azithromycin resistance from the results of erythromycin sensitivity testing using breakpoints defined by the Clinical and Laboratory Standards Institute [32]. We report sensitivity results for (1) *S. aureus* and (2) pyogenic streptococci (groups A, C, and G) collectively, including *S. pyogenes* (group A).

*Emm*-typing was performed according to the protocol specified by the Centers for Disease Control and Prevention with minor modifications, as previously described [33]. *Emm*-clusters were deduced based on the *emm*-typing results [34].

### Statistical Analysis

The study was designed to assess whether adding a single oral dose of azithromycin, alongside ivermectin, resulted in a decrease in the prevalence of impetigo at 12 months compared to treatment with ivermectin alone. We calculated the prevalence of scabies and impetigo in each study arm at baseline and 12 months. We calculated the absolute and relative reduction in scabies and impetigo prevalence between baseline and 12 months. We compared the change in prevalence, separately for scabies and impetigo, between study groups by calculating the ratio of the prevalence at baseline and 12 months for each group, and testing the hypothesis that these 2 ratios were equal [35].

### Sample Size Calculations

We estimated the pre-MDA prevalence of scabies and impetigo to be approximately 15% and 25% respectively. Based on previous studies, we anticipated the prevalence of scabies would fall to 1% in both arms and the prevalence of impetigo would fall to 10% at 12 months in the ivermectin-only arm [11, 19]. Assuming that in the combined-treatment arm impetigo prevalence fell to 5%, and loss-to-follow-up was 10%, we needed to enroll 635 individuals in each study arm to have 80% power to detect a difference between study arms as significant at the 0.05 level. As a secondary outcome we calculated the proportion of *S. aureus* and *S. pyogenes* isolates which were macrolide resistant in each arm at baseline, 3 months and 12 months. Statistical analysis was conducted in R 3.4.2 [36].

### Ethics Approval

The study was approved by the London School of Hygiene & Tropical Medicine, the Solomon Islands National Health Ethics Committee and the Atoifi Adventist Hospital Ethics Committee. Azithromycin was provided by WHO (who purchased it from Medopharm [India]). Ivermectin was purchased from Merck Sharp and Dohme (Australia). Permethrin was purchased from Pharmatec (Fiji). At 12 months, all individuals in the ivermectin-only arm were offered azithromycin in line with WHO guidelines for the treatment of yaws [13]. The study was prospectively registered on clinicaltrials.gov (NCT02775617). All authors had access to study data and shared responsibility for the decision to submit for publication.

## RESULTS

At baseline, 1291 individuals (90.8% of the resident population in the 6 study communities) were examined and received treatment. At the 12-month follow-up the resident population of the study communities had decreased to 1255, of whom 1083 individuals were examined (86.3%) (Table 1). Follow-up was lower in the ivermectin-only arm at 12 months (ivermectin-only 76.2% vs combined treatment arm 96.3%). Overall, 46.6% of participants were male, and the median age of participants was 25 years (IQR 11–47) (Table 1).

**Table 1.** Demographics

	Ivermectin Only Arm		Combined Treatment Arm	
	Baseline	12 Months Follow-up	Baseline	12 Months Follow-up
Resident population	717	627	705	628
Enrolled population (%)	638 (88.9%)	478 (76.2%)	653 (92.6%)	605 (96.3%)
Median age [inter-quartile range] (years)	25 [12–47]	25 [10–47]	24 [10–45]	26 [12–47]
Sex (male) (%)	326 (51.1%)	212 (44.4%)	318 (48.7%)	297 (49.1%)

At baseline the prevalence of scabies was 11.8% (95% confidence interval [CI] 9.4–14.6%) in the ivermectin-only arm and 9.2% (95% CI 7.1–11.7%) in the combined-treatment arm. The severity of scabies was similar in both arms; overall, 77.8% of individuals had mild scabies, 20% had moderate scabies, and 2.2% had severe scabies (data not shown). No cases of crusted scabies were detected. At baseline the prevalence of active impetigo was 10.1% (95% CI 8.1–13.0%) in the ivermectin-only treatment arm and 12.1% (95% CI 9.7–14.9%) in the combined-treatment arm. The severity of impetigo was similar in both groups; overall, 84.1% of participants had mild impetigo, 11% had moderate impetigo, and 4.9% had severe impetigo (data not shown).

At 12 months the prevalence of scabies and impetigo had fallen to 1.0% (95% CI 0.3–2.6%) and 2.5% (95% CI 1.4–4.5%), respectively, in the ivermectin-only treatment arm and to 0.7% (95% CI 0.2–1.8%) and 3.3% (95% CI 2.1–5.1%), respectively, in the combined treatment arms (Table 2). There was no significant difference between the two groups (91.5% vs 92.4%, *P* = .31), in the change from baseline to 12 months in scabies prevalence or the change in impetigo prevalence (75.2% vs 72.7%, *P* = .49).

**Table 2.** Prevalence of Scabies and Impetigo

		Baseline	12 Months	Absolute Reduction	Relative Reduction
Ivermectin only	Scabies	11.8% (95% CI 9.4–14.6%) (n = 75/638)	1.0% (95% CI 0.3–2.6%) (n = 5/478)	10.8% (95% CI 8.0–13.4%)	91.5% (95% CI 68.5–100%)
	Impetigo	10.1% (95% CI 8.1–13.0%) (n = 66/638)	2.5% (95% CI 1.4–4.5%) (n = 12/478)	7.6% (95% CI 5.1–10.6%)	75.2% (95% CI 67.9–100%)
Combined treatment	Scabies	9.2% (95% CI 7.1–11.7%) (n = 60/653)	0.7% (95% CI 0.2–1.8%) (n = 4/605)	8.5% (95% CI 6.2–10.8%)	92.4% (95% CI 49.2–100%)
	Impetigo	12.1% (95% CI 9.7%–14.9) (n = 79/653)	3.3% (95% CI 2.1–5.1%) (n = 20/605)	8.8% (95% CI 5.9–11.7%)	72.7% (95% CI 48.9–96.5%)

Abbreviation: CI, confidence interval.

We performed a post hoc sensitivity analysis to assess whether the lower follow-up in the ivermectin-only arm might have affected our results. We calculated the prevalence of impetigo that would have been seen in the ivermectin-only treatment arm if we had achieved a follow-up at a level similar to the combined treatment arm and the prevalence amongst participants not seen at 12 months had been unchanged from baseline. Under these assumptions, the prevalence of impetigo in the ivermectin-only treatment arm would have been 4.1% at twelve months. In this analysis there was no significant difference in the relative reduction in impetigo between arms (60.2% vs 72.7%, *P* = .23).

Swabs were collected from 73 people with impetigo at baseline, 36 people at 3 months, and 22 people at 12 months. At baseline, 80% of impetigo lesions from which we obtained a swab yielded *S. aureus* on culture and 62% yielded pyogenic streptococci (predominantly *S. pyogenes,* 56%). At 3 and 12 months the proportion of *S. aureus* was unchanged (78% and 86%, respectively), but the proportion of impetigo lesions from which *S. pyogenes* were cultured had fallen significantly to 33% at 3 months (*P* = .04 for the comparison to baseline) and 23% at 12 months (*P* < .01 for the comparison to baseline). The relative decrease in *S. pyogenes* was similar in both arms of the study (Table 3).

**Table 3.** Impetigo Culture Results

		Baseline	3 Months	12 Months
		Organism Isolated (%, 95% CI)	Organism Isolated (%, 95% CI)	Organism Isolated (%, 95% CI)
Ivermectin only	*Staphylococcus aureus*	27/35 (77%, 59–89%)	13/19 (68%, 43–86%)	6/6 (100%, 52–100%)
	Pyogenic streptococci^a^	28/35^b^ (80%, 63–91%)	9/19^c^ (47%, 25–71%)	1/6 (17%, 9–64%)
Combined treatment	*Staphylococcus aureus*	32/38 (84%, 68–93%)	15/17 (88%, 62–98%)	13/16 (81%,54–95%)
	Pyogenic streptococci^a^	17/38^b^ (45%, 29–63%)	5/17^c^ (29%, 11–56%)	4/16 (25%, 8–53%)
Total	*Staphylococcus aureus*	59/73 (81%, 70–89%)	28/36 (78%, 60–89%)	19/22 (86%, 64–96%)
	Pyogenic streptococci^a^	45/73 (62%, 49–73%)	14/36 (39%, 24–56%)	5/22 (23%, 9–46%)

Abbreviation: CI, confidence interval.

^a^For simplicity we report Group C/G streptococci alongside *Streptococcus pyogenes*.

^b^One group C/G streptococcus in the ivermectin-only treatment arm and 4 in the combined treatment arm.

^c^Two group C/G streptococcus in the ivermectin-only treatment arm.

No macrolide resistance was detected among streptococci in either arm at any of the 3 time points. In the ivermectin-only treatment arm we did not isolate any macrolide-resistant *S. aureus* at any time point. In the combined-treatment arm, one isolate of *S. aureus* was macrolide-resistant at baseline, and 8/15 (53%) of *S. aureus* isolates were macrolide-resistant at 3 months. At 12 months, no macrolide-resistance was detected in any of the 6 isolates tested (Table 4). Isolates of *S. pyogenes* fell into 27 different *emm*-types. Twenty-five *emm*-types could be categorized into one of 11 different *emm*-clusters (Supplementary Table 1).

**Table 4.** Antimicrobial Sensitivity Testing Results

		Baseline	3 Month	12 Months
		Macrolide Resistant (%, 95% CI)	Macrolide Resistant (%, 95% CI)	Macrolide Resistant (%, 95% CI)
Ivermectin only	*Staphylococcus aureus*	0/27 (0%, 0–16%)	0/13 (0%, 0–28%)	0/6 (0%, 0–48%)
	Pyogenic streptococci^a^	0/30 (0%, 0–14%)	0/9 (0%, 0–37%)	0/1 (0%, 0–95%)
Combined treatment	*Staphylococcus aureus*	1/32 (3%, 0.2–18%)	8/15 (53%, 27–78%)	0/13 (0%, 0–28%)
	Pyogenic streptococci^a^	0/19 (0%, 0–21%)	0/5 (0%, 0–54%)	0/4 (0%, 0–60%)
Total	*Staphylococcus aureus*	1/59 (1.7%, 0–10%)	8/28 (29%, 14–49%)	0/19 (0%, 0–21%)
	Pyogenic streptococci^a^	0/45 (0%, 0–13%)	0/14 (0%, 0–27%)	0/5 (0%, 0–54%)

Abbreviation: CI, confidence interval.

^a^For simplicity we report Group C/G streptococci alongside *S. pyogenes.*

## CONCLUSION

In the first study to directly compare co-administration of azithromycin and ivermectin with ivermectin-only MDA, co-administration did not result in a greater decrease in the clinical prevalence of impetigo at 12 months, compared to ivermectin alone. Substantial decreases were observed in both the prevalence of scabies and impetigo, but the magnitude of the decrease was similar in the 2 study arms and consistent with the effect size seen in previous studies [11]. In both arms, we observed a large reduction in the proportion of impetigo lesions from which pyogenic streptococci were isolated, whereas the proportion of lesions from which *S. aureus* was cultured did not change in either arm.

A major aim of scabies control programmes is a reduction in sequelae of *S. pyogenes* infection. Our study provides some of the first data demonstrating that the observed reduction in clinical impetigo may be due to a reduction in *S. pyogenes* infection. This decrease in pyogenic streptococci occurred in both communities that received ivermectin alone and those in which it was co-administered with azithromycin. Why *S. pyogenes* should decline to a greater extent than *S. aureus* is unclear. Asymptomatic carriage of *S. aureus* is more common than carriage of *S.pyogenes* and can persist following MDA with azithromycin [17], so it might serve as a potential reservoir for ongoing transmission. Our data do not allow us to assess this hypothesis, and future studies to better understand the impact of MDA on impetigo lesions are warranted.

We observed an increase at 3 months in the proportion of strains of *S. aureus* that were macrolide resistant following MDA with azithromycin. This effect appeared to wane by 12 months post-MDA, although our sample size was too small to draw a firm conclusion on the duration of the effect. In the communities studied, there is limited use of macrolides other than in the management of sexually transmitted infections. The lack of ongoing selective pressure may have contributed to the return to a wild-type antibiotic susceptibility pattern at 12 months. Previous studies have demonstrated transient increases in the nasopharyngeal carriage of azithromycin resistant *Streptococcus pneumoniae* following azithromycin MDA, with limited evidence that multiple rounds of MDA lead to greater selection of resistant isolates than a single round [14–16]. A study of nasopharyngeal carriage of *S. aureus* found macrolide resistance increased within a month of azithromycin MDA but then declined over 6 months. Individuals who received multiple rounds of MDA were more likely to have resistant strains than those who had received only one round [17]. Collectively, these data highlight the need for ongoing vigilance concerning the impact of azithromycin MDA on organisms other than those that are the immediate target but also suggest that infrequent (annual) MDA of azithromycin is unlikely to substantially affect macrolide resistance rates in Gram-positive organisms [18].

Our study has several limitations. First, and consistent with other studies assessing the impact of MDA, it was not blinded. Second, the diagnosis of scabies and impetigo was made on clinical grounds alone, albeit by a single experienced physician using criteria that have previously been shown to have good sensitivity and specificity [27]. Third, follow-up rates differed between our 2 study arms. In one village in the ivermectin-only treatment arm, rumors circulated that MDA was being conducted without approval from the local hospital even though hospital staff made up the majority of the field-team. Meetings were held with community leaders and the study team including the hospital Director of Nursing (RA), but follow-up in this village remained lower than other villages in the study. Despite this, we had an adequate sample size to demonstrate that there was no additional reduction in impetigo prevalence in the arm receiving combined treatment, and our sensitivity analysis was consistent with our overall results. Fourth, we did not collect swabs from all individuals with active impetigo (nor from every lesion on individuals with multiple lesions). We cannot exclude the possibility that increasing the proportion of individuals from whom swabs were collected might have altered the proportion of samples containing pyogenic streptococci or macrolide-resistant bacteria. Finally, samples were shipped to Australia, a journey that might also have reduced our pathogen recovery rate. However, we successfully isolated *S. aureus,* a streptococcus, or both from more than 95% of swabs so think it unlikely that the transport process affected our results. Our results are consistent with previous studies on changing patterns of carriage of antimicrobial resistant flora following MDA and provide some of the first bacteriological endpoint data on the impact of ivermectin MDA on impetigo.

Our data add to those from a small number of previous studies examining the potential of combining individual MDA programmes into a single intervention. Our study did not aim to investigate the safety of co-administration of ivermectin and azithromycin, as existing pharmacokinetic and trial data already support the safety of co-administration of these agents [25, 26], and we have recently completed a large scale field study directly addressing the question of safety at a district-level (ACTRN12613000474752) [37]. Although we were unable to detect any clinical impact on impetigo prevalence of adding azithromycin to ivermectin MDA on impetigo prevalence, co-administration still has potential logistical and financial benefits by treating multiple NTDs via a single intervention. Further studies on integrated approaches are needed to draw firmer conclusions about the potential benefit on disease occurrence.

Ivermectin MDA has emerged as a central component of the control strategy for scabies in high prevalence communities. Our data suggest the addition of a single dose of azithromycin, at a single timepoint, neither translates to an additional benefit in reducing impetigo prevalence at 12 months nor results in an increased prevalence of antimicrobial resistance. It is not known whether alternative strategies, such as biannual MDA or use of an alternative antimicrobial agent, might be more successful. Further investigation may help to optimize community interventions for the control of scabies and its sequelae.

## Supplementary Data

Supplementary materials are available at *Clinical Infectious Diseases* online. Consisting of data provided by the authors to benefit the reader, the posted materials are not copyedited and are the sole responsibility of the authors, so questions or comments should be addressed to the corresponding author.

Supplementary Table 1

## Notes

***Contributors.*** M. M. wrote the first draft of the paper. C. B., K. A., and E. P. conducted laboratory work. M. M., H. T., C. K., J. A., and R. A. conducted fieldwork. M. M., C. B., and K. A. analyzed the data. M. M., J. D., J. K. M., L. R., M. R. M, D. M., A. W. S., D. C. W. M., and A. S. designed and supervised the study. All authors revised the manuscript.

***Financial support.*** M. M. was supported by a Wellcome Trust Clinical PhD fellowship (102807).

***Potential conflicts of interest.*** J. D. reports grants from the International Trachoma Initiative. M. M. reports consultancy fees from the World Health Organization. All other authors report no potential conflicts of interest. All authors have submitted the ICMJE Form for Disclosure of Potential Conflicts of Interest. Conflicts that the editors consider relevant to the content of the manuscript have been disclosed.
