# Supplementary material for: Randomized Trial of Community Treatment With Azithromycin and Ivermectin Mass Drug Administration for Control of Scabies and Impetigo
Source: Clin Infect Dis. 2018 Jul 7;68(6):927–33. doi: 10.1093/cid/ciy574 (PMC6399435; doi:10.1093/cid/ciy574)
Supplement: Supplementary Table 1 [file ciy574_suppl_supplementary_table_1.docx]

**Supplementary Table 1: *Emm-*typing of Group A Streptococcal isolates**

| ***emm*-type** | ***emm*-cluster** | **Number of Samples** |
| --- | --- | --- |
| **36** | **D1** | **4** |
| **100** | **D2** | **1** |
| **116** | **D4** | **3** |
| **52** | **D4** | **7** |
| **53** | **D4** | **1** |
| **70** | **D4** | **1** |
| **86** | **D4** | **1** |
| **108** | **D4** | **1** |
| **165** | **E1** | **1** |
| **104** | **E2** | **2** |
| **106** | **E2** | **1** |
| **68** | **E2** | **1** |
| **92** | **E2** | **2** |
| **209** | **E3** | **3** |
| **44** | **E3** | **2** |
| **9** | **E3** | **1** |
| **102** | **E4** | **1** |
| **77** | **E4** | **1** |
| **109** | **E4** | **1** |
| **169** | **E4** | **1** |
| **63** | **E6** | **1** |
| **105** | **Clade Y** | **1** |
| **57** | **M57** | **2** |
| **222** | **M222** | **4** |
| **STG 653** | **N/A** | **13** |
| **162** | **N/A** | **1** |
